# Whole-cell patch clamp and extracellular electrophysiology recordings in mouse brain slices

**DOI:** 10.1016/j.xpro.2025.104008

**Published:** 2025-07-31

**Authors:** Huimin Chen, Xiangyi Shi, Fuzheng Guo

**Affiliations:** 1 Department of Neurology, School of Medicine, University of California, Davis, Sacramento, CA 95817, USA; 2 Institute of Pediatric Regenerative Medicine (IPRM), Shriners Hospitals for Children – Northern California, Sacramento, CA 95817, USA

**Keywords:** Cell Biology, Model Organisms, Neuroscience

## Abstract

Electrophysiological recordings enable the assessment of neuronal excitability, synaptic function, and network activity. Here, we present a protocol for whole-cell patch clamp and extracellular electrophysiology recordings in mouse brain slices. We describe steps for acute brain slice preparation, whole-cell recordings from neurons and astrocytes, and extracellular recordings of compound action potentials and long-term potentiation. We detail procedures for solution preparation, data acquisition, and analysis, with troubleshooting strategies to ensure experimental reliability and reproducibility.

For complete details on the use and execution of this protocol, please refer to Wang et al.^1^

## Highlights

• Steps for preparing live brain slices and extracellular and intracellular solutions
• Instructions for whole-cell patch clamp of pyramidal neurons and astrocytes
• Procedures for extracellular recording of CAPs and LTP
• Guidance on data analysis of electrophysiology recordings

## Abstract (editor highlights)

Publisher’s note: Undertaking any experimental protocol requires adherence to local institutional guidelines for laboratory safety and ethics.

## Graphical abstract

**Figure:**
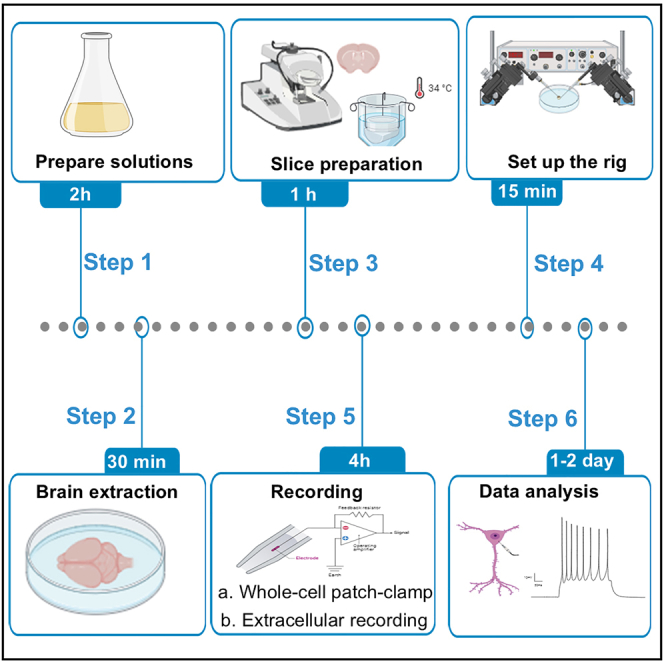

## Before you begin

Electrophysiology provides a powerful approach for investigating the electrical properties of neurons and glial cells by enabling direct measurements of membrane potentials, synaptic currents, and network activity. However, its complexity demands extensive training, precise execution, and specialized expertise, which can make it challenging to obtain reliable and reproducible results.

**This protocol introduces several conceptual and methodological improvements over existing patch-clamp techniques. First**, this protocol offers specific adjustments to intra- and extracellular solutions to optimize different types of recordings. For example, we typically maintain extracellular KCl at 2.5 mM to assess basic membrane properties and spiking. Increasing the concentration to 3.0 mM can modestly enhance neuronal excitability and promote spontaneous synaptic release, particularly of excitatory neurotransmitters. **Second**, we outline detailed brain slicing procedures—including ice-cold perfusion, use of crushed ice during dissection, rapid processing, and application of glycerol/vitamin C-enriched solutions—to maximize tissue viability, reduce oxidative stress and improve slice quality and recording consistency. **Third**, the protocol combines whole-cell and extracellular recordings (e.g., LTP and CAP) to facilitate a more intuitive understanding of electrophysiological principles at both the cellular and network levels. Representative data and troubleshooting tips further support hands-on learning and help users successfully carry out their own recordings. In summary, the integration of whole-cell and extracellular recording techniques not only supports a range of experimental paradigms but also enables new users to grasp the fundamentals of whole-cell recordings more clearly through direct comparison with extracellular signals. To ensure smooth progression of subsequent steps, researchers should prepare both extracellular and intracellular solutions in advance.

We optimized this protocol for both novice and experienced electrophysiologists, making it applicable to a wide range of basic neuroscience studies and disease models. In this protocol, we target both neurons and glial cells for patch clamp recordings. Rapid and accurate identification of cell types under microscopes plays a critical role in data collection and interpretation. We recognize neurons by their larger soma and excitable membranes that fire action potentials in response to depolarizing current injections.^2^^,^^3^ Among them, pyramidal neurons exhibit triangular-shaped soma and a prominent apical dendrite (Figure 2C), whereas medium spiny neurons have smaller, rounder somas with dense dendritic spines and inward rectification at hyperpolarizing potentials.^4^^,^^5^ In contrast, most mature glial cells such as astrocytes and differentiated oligodendrocyte exhibit passive membrane properties and do not fire action potentials.^6^^,^^7^ Identify astrocytes by their selective labeling with SR101 following a 20-min incubation (Figure 4C). However, prolonged incubation times (e.g., 40 min or more) can cause non-specific labeling of oligodendrocytes, as demonstrated by multiple publications.^8^^,^^9^^,^^10^^,^^11^ Researchers also alternatively identify astrocyte with their morphology characteristic under DIC optics. Oligodendrocyte precursor cells (OPCs), also known as NG2 glia, possess voltage-gated conductances despite not firing action potentials. Researchers historically referred to these cells as “complex glia” due to their intricate current.^12^^,^^13^^,^^14^ Identify OPCs using transgenic fluorescent reporter lines, such as NG2-DsRed, where the fluorescent protein expression is driven by the NG2 (Cspg4) promoter.^15^ These distinctions guide targeted recordings in the following steps.

### Institutional permissions

The Institutional Animal Care and Use of Committee at the University of California, Davis approved all mouse procedures. We use postnatal 25 days (PN25) C57BL/6 mice for whole cell patch clamp recordings and 3-month-old C57BL/6 mice for extracellular recordings, including CAP and LTP measurements. Researchers will need to acquire permissions from the relevant institutions.

### Solution preparation

**Timing: 2 h**

We optimize solution conditions to maintain cell viability and achieve stable, long-term electrophysiological recordings. Maintaining cell integrity is particularly critical for successful whole-cell recordings in ex vivo brain slices. We modify the recipe of the solutions used during brain slice cutting, incubating and recording. To minimize excitotoxicity during tissue sectioning, we utilize a **cutting solution** containing glycerol, low calcium, and high magnesium, which helps sustain neuronal health throughout the slicing process.^16^^,^^17^^,^^18^ After sectioning, we transfer brain slices into **artificial cerebrospinal fluid (aCSF)** with proper oxygenation and pH balance to maintain physiological conditions. We carefully adjust the potassium concentration in aCSF because it plays a crucial role in neuronal excitability and enhances synaptic activity.^19^ For astrocyte recordings, we maintain low K^+^ and control Ca^2+^/Mg^2+^ levels to prevent excessive depolarization and preserve a stable resting membrane potential.^20^^,^^21^^,^^22^ Additionally, we incorporate ascorbic acid to mitigate oxidative stress, thereby preserving tissue integrity for extended experimental durations.

We use the cutting solution to section brain slice. It contains (in mM): 220 glycerol, 2.5 KCl, 1.25 NaH_2_PO_4_·H_2_O, 25 NaHCO_3_, 0.5 CaCl_2_· 2H_2_O, 7 MgCl_2_·6H_2_O, and 20 D-glucose.^23^ Use the cutting solution to minimize tissue damage and maintain cell viability. Low calcium and high magnesium concentration (0.5 mM Ca^2+^, 7 mM Mg^2+^) reduces calcium-dependent excitotoxicity, minimizes tissue damage, and improves survival rates. Glycerol substitutes traditional sucrose for more effective membrane protection and enhances the physiological quality of brain slices.^17^^,^^18^

We use artificial cerebrospinal fluid solution (aCSF) for slice incubation and recording. It contains (in mM): 125 NaCl, 2.5 KCl, 25 NaHCO_3_, 1.25 NaH_2_PO_4_· H_2_O, 2.5 CaCl_2_· 2 H_2_O, 1.3 MgCl_2_·6H_2_O, and 10 D-glucose. Continuously bubble the aCSF with 95% O_2_ and 5% CO_2_ to maintain oxygenation and pH balance. Use normal aCSF to investigate neuron and astrocyte properties, as well as neuronal spiking. Restoration of Ca^2+^ and Mg^2+^ to near-physiological levels (2.5 mM Ca^2+^, 1.3 mM Mg^2+^) supports synaptic transmission. Prepare 1 L solutions of cutting solution and aCSF, and store for up to one week at 4°C. We include ascorbic acid (vitamin C, 0.4 mM) in both cutting solution and aCSF to reduce oxidative stress, protect brain slices, and support long-term experiments.

***Note:*** Reduce the concentration of CaCl_2_ to approximately 1.5 mM during astrocyte recordings to minimize synaptic overactivation and excitotoxicity.^20^^,^^24^

**CRITICAL:** When recording spontaneous EPSCs, increasing the extracellular KCl concentration of aCSF to 3.0–3.5 mM or higher raises the extracellular potassium ion concentration. This leads to slight depolarization of the resting membrane potential, enhancing neuronal excitability and spontaneous synaptic activity.^19^

We use two types of intracellular solutions specifically formulated for different recording modes to optimize signal quality and ensure experimental reliability. Adjust the pH of the intracellular solution to 7.25 with KOH/HCl (pH 7.25, 280–290 mOsm), aliquot it, and store at −20°C. Filter it with a 0.2 μm filter using a 1 mL syringe before use. This optimized solution ensures precise and reliable electrophysiological measurements.

We use a KCl-based intracellular solution containing (in mM): 135 KCl, 0.5 EGTA, 10 HEPES, 2 Mg-ATP, 0.2 Na-GTP, and 4 Na_2_-phosphocreatine^23^ to record action potentials and spontaneous inhibitory postsynaptic currents (sIPSCs). The high KCl concentration minimizes liquid junction potentials, prevents interference from the K^+^ reversal potential, and preserves neuronal excitability during action potential and passive electrophysiological recordings. We add 6,7-dinitro-quinoxaline-2,3-dione (DNQX; 10 μM) and d,l-2-amino-5-phosphonovalerate (AP5; 20 μM) to the extracellular solution to block ionotropic glutamate receptors to record IPSCs.

We record mIPSCs^17^ using a CsCl-based solution containing (in mM): 130 CsCl, 5 KCl, 0.5 EGTA, 10 HEPES, 2 Mg-ATP, 0.2 Na-GTP, 4 Na_2_-phosphocreatine with pH 7.25 and 280–290 mOsm. To record EPSCs (including sEPSCs, mEPSCs, and evoked EPSC) in voltage-clamp mode,^23^ we use a modified version of this solution in which we replace half of the CsCl with KCl, resulting in a final composition of 67.5 mM CsCl and 67.5 mM KCl. When recording evoked complex EPSCs and mEPSCs, we add the Na^+^ channel blocker lidocaine *N*-ethyl bromide (QX-314, 4 mM) to the intracellular solution to block Na spikes.^25^ Add Cs^+^ in the internal solution to non-selectively block inwardly rectifying (Kir) and voltage-gated (Kv) potassium channels, thereby suppressing K^+^ currents and improving the resolution of synaptic events, minimizing K^+^ currents.

We performed all EPSC and IPSC recordings at a holding potential of −70 mV. We add tetrodotoxin (TTX, 1 μM) to the extracellular solution to isolate mIPSCs and mEPSC, and add picrotoxin (100 μM) to block GABA_A_ receptors during EPSC recordings.

***Note:*** Picrotoxin can induce network hyperexcitability and epileptiform activity. Previous studies have shown that bath-applied picrotoxin (50–200 μM) induces paroxysmal depolarizing shifts and epileptiform bursting in CA3/CA1 hippocampal regions.^26^ We applied picrotoxin only when necessary and carefully interpreted the data obtained under these conditions. If needed, physically isolate the recording region to further reduce recurrent activity, depending on the experimental design.

Refer to Table 1 for a comparative summary of the intracellular solutions and their application-specific advantages. Select the appropriate solution based on experimental goals, as this strategic choice optimizes synaptic event detection and facilitates a mechanistic understanding of neuronal communication across different electrophysiological modalities.

**Table 1:** Intracellular solutions for whole-cell patch-clamp recording

Solution type	Major Component(s)	Chloride level	Advantages	Application
K-gluconate	K- gluconate	Low	Low intracellular Cl^-^ shifts E_Cl to ∼ −70 mV, weakening IPSCs. EPSCs and IPSCs are distinguishable at 0 mV.	liquid junction potentials (LJP) ∼10 mV; gluconate may block channels and is suboptimal for IPSCs.
Cs-gluconate	Cs^+^ blocks most K^+^ channel			
K-methanesulfonate	K-methanesulfonate			Compared with gluconate, better preserves neuronal excitability and reduces depletion of calcium-activated potassium currents.
Cs-methanesulfonate	Cs^+^ blocks most K^+^ channel			
KCl-based	KCl	High	E_Cl near 0 mV. The inward IPSCs (easy to detect) at -70mV. Small LJP errors.	Recording action potential and sIPSC.
CsCl	Cs^+^ blocks most K^+^ channel			Not suitable for studying excitability or K^+^ channels; good for recording miniature/evoked IPSCs.
CsCl+KCl	130 mM CsCl and 5 mM KCl; or a 1:1 mixture of CsCl and KCl; blocks most K^+^ channel			Improves cell health and stability; good for recording mIPSCs and EPSCs.

## Key resources table

**Table:** 

REAGENT or RESOURCE	SOURCE	IDENTIFIER
**Chemicals, peptides, and recombinant proteins**		

SR101	Sigma-Aldrich	Cat# S7635
Tetrodotoxin	Hello Bio	Cat# HB1035
Ox-314	Sigma-Aldrich	Cat# 552233
Gabazine	Sigma-Aldrich	Cat# S106
DL-AP5	Abcam	Cat# ab120004
CNQX	Sigma-Aldrich	Cat# C239
Glycerol	Acros	Cat# 15892-0025
NaH_2_PO_4_·H_2_O	Sigma-Aldrich	Cat# 71505
NaHCO_3_	Sigma-Aldrich	Cat# S5761
CaCl_2_· 2H_2_O	Sigma-Aldrich	Cat# C5080
MgCl_2_·6H_2_O	Sigma-Aldrich	Cat# M0250
D-glucose	Sigma-Aldrich	Cat# SLCQ7550
KCl	Sigma-Aldrich	Cat# 60128
NaCl	Sigma-Aldrich	Cat# S7653
L-Ascorbic acid	Sigma-Aldrich	Cat# A0278
EGTA	Sigma-Aldrich	Cat# E−3889
HEPES	Sigma-Aldrich	Cat# 54457
Mg-ATP	Sigma-Aldrich	Cat# A-9187
Na-GTP	Sigma-Aldrich	Cat# G-8877
Na_2_-phosphocreatine	Sigma-Aldrich	Cat# P-7936
CsCl	Sigma-Aldrich	Cat# C-4036

**Software and algorithms**		

Patch Clamp Data Acquisition and Analysis	Molecular Devices	pClamp
Origin Graphing and Analysis Software	OriginLab Corporation	Origin 2018
Mini data analyzing software	Synaptosoft	Mini Analysis

**Other**		

Patch-clamp amplifier	Molecular Devices	MultiClamp 700B
Digitizer	Molecular Devices	Digidata 1550B
Fluorescence microscope system	Nikon	Nikon Eclipse e600FN
Electric Micro-manipulator	Sutter	MP225
Peri-Star Pro pump	World Precision Instruments (WPI)	LEAD15-44
Vibrating microtome	Leica	VT1000S
Micropipette puller	Narishige	PC-10
Temperature controller	Warner Instruments	TC-344C
Glass pipette	Sutter	B150-110-10

## Step-by-step method details

### Preparation work before cutting brain slices

**Timing: 20 min**

**Figure 1:**
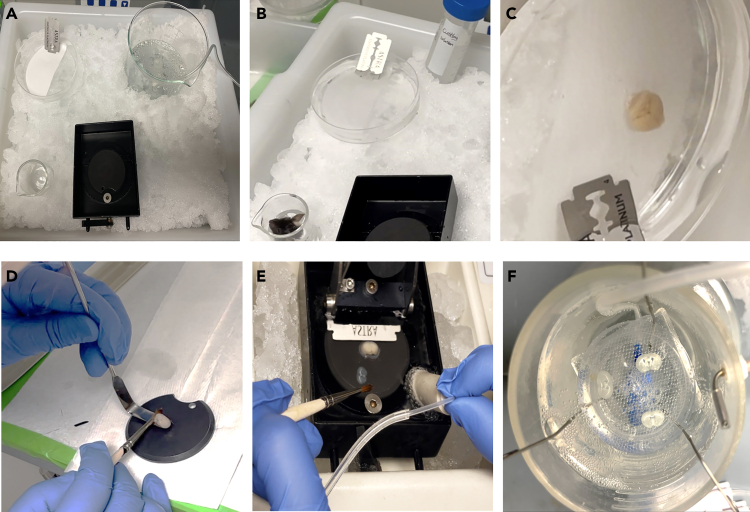
Mouse brain slices preparation (A) Pre-cool cutting solutions and tools. (B) Immerse mouse brain into cold cutting solution. (C) Extracted brain in the dish with cold cutting solution. (D) Glue the brain on the vibratome plate. (E) Cutting coronal sections with a Leica microtome. (F) Brain slice in an incubation chamber (34°C) bulled with mixed oxygen.
  b. Prepare dissection tools and perfusion setup.
    i. Prepare the following tools: a fine-tipped brush, surgical scissors, broad-tip tweezers, and a long-handled tissue transfer tool with one curved and one straight end (for stable brain transfer).
    ii. Prepare a syringe for perfusion and draw 30 mL of cold cutting solution before use.
      ***Note:*** These steps maintain low temperatures during slicing, which helps obtain high-quality brain slices for whole-cell recordings.
  c. Prepare incubation solution.
    i. Add ∼300 mL of 4°C **aCSF** to the incubation beaker, ensuring it covers the incubation chamber filter (Brain Slice Incubators, S-BSK1, Automate Scientific).
    ii. Place it in a 34°C water bath, keeping the water bath level equal to the incubation solution.
    iii. Continuously bubble with 95% O_2_/5% CO_2_.

1. Preparation work before euthanizing mice.
  a. Set up cold environment and tools for brain trimming.
    i. Pre-freeze 30 mL of cutting solution at −20°C for cutting slices.
    ii. Fill a large ice box with crushed ice.
    iii. Place a 500 mL beaker on the ice and pour in 300 mL of **cutting solution** (stored at 4°C), continuously bubbled with 95% O_2_ and 5% CO_2_.
    iv. Place a petri dish with a scalpel blade and filter paper for brain trimming on the ice.
    v. Insert an empty 25 mL beaker into the ice for later brain immersion (Figure 1A).

### Brain slice preparation

**Timing: 20 min**

2. Slice preparation.
  a. Anesthetize the mouse using a ketamine/xylazine mixture.
  b. Secure the mouse on the perfusion tray.
  c. Perform transcardial perfusion by injecting approximately 25 mL ice-cold cutting solution via the right ventricle.
  d. Quickly dissect the head immediately after perfusion.
  e. Immerse the head in oxygenated ice-cold cutting solution for 5 s to cool down the brain before brain dissection (Figure 1B).
  f. Quickly and carefully extract the brain to avoid tissue damage.
  g. Trim the brain on a petri dish filled with cutting solution coronal or horizontally depending on experimental needs and requirements (Figure 1C).
  h. Stabilize the trimmed brain onto the support plate of the cutting chamber for vibratome sectioning. We recommend using the “502 super-glue” for brain stabilization (Figure 1D).
    ***Note:*** Apply an appropriate amount of the glue to secure the brain during slicing. Excess glue can damage the tissue, whereas insufficient glue may lead to brain movement or detachment.
  i. Cut coronal 300 μm brain slices using a Leica VT1000S vibratome (Leica Microsystems, Germany).
  j. Secure the bottom plate in the clamping slot and wait 2–3 s for the glue to dry.
  k. Install the blade with a ∼10° tilt.
  l. Pour pre-oxygenated cutting solution into the slicing chamber.
    ***Note:*** Avoid direct contact with the brain to prevent displacement, and ensure that the cutting solution fully submerges both the brain and the blade.
  m. Place crushed ice around the slicing chamber to maintain low temperatures (Figure 1E).
  n. Add −20°C iced cutting solution to create an ice-water mixture for rapid cooling.
  o. Adjust the vibration frequency, slicing speed, and start/stop positions for continuous slicing.
  p. Use a brush or pipette to gently collect the slices while continuously bubbling oxygen (Figure 1E).
  q. Carefully transfer brain slices onto the mesh in the incubation chamber (Figure 1F).
  r. Adjust gas flow to prevent slices from drifting while maintaining sufficient oxygenation for viability.
    ***Note:*** Ensure no bubbles remain on the slices for proper settling.
  s. Maintain the holding chamber at 34°C for 30 min, then keep at room temperature (23°C).
    ***Note:*** Perfuse the mouse with ice-cold cutting solution before brain extraction and keep all solutions on ice to preserve the physiological state and neuronal activity of the brain slices.
    **CRITICAL:** The brain consumes large amounts of oxygen and nutrients to sustain its high metabolic activity. After decapitation, the loss of oxygen supply rapidly depletes energy stores and causes neuronal damage. To mitigate this, we use ice-cold cutting solution (∼0°C–4°C) to slow down enzymatic activity and metabolic processes, thereby reducing ATP consumption and preventing ischemic injury.

### Patch-clamp rig setup

**Timing: 15 min**

**Figure 2:**
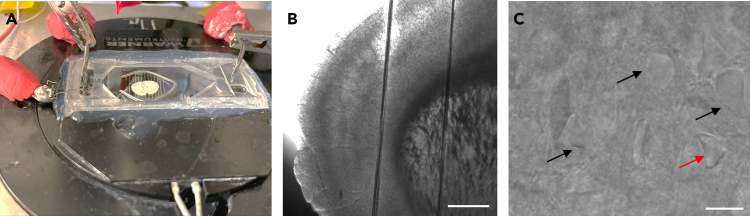
Selecting healthy neurons for whole-cell patch-clamp recording (A) Brain slice in the recording chamber. (B) A low power microphotograph showing a coronal brain slice. Scale bar represents 0.5 mm. (C) 60× magnification image showing a healthy cortical pyramidal neuron. Black arrows in C indicate healthy neurons; and red arrow points to a damaged neuron. Scale bar represents 15 μm.
  e. Secure the slice in place using a slice anchor (pressing grid) (Warner instruments, Slice Anchors & Kits) (Figure 2B).
    ***Note:*** Adjust the slice and grid position with forceps, ensuring the target region is within the grid openings.
  f. Locating the target cells.
    i. Use a low-magnification objective to find the target brain region.
    ii. Switch to a 60× objective and focus on individual cells for patch-clamp recording (Figure 2C).

3. Preparation work.
  a. Continuously perfuse the aCSF with 95% O_2_ and 5% CO_2_ with the pump (WPI, Peri-star Pro pump).
    ***Note:*** Ensure that the solution circulates through the recording chamber (30°C–32°C) at a stable flow rate (2 mL/min). For continuous perfusion during recording, gravity perfusion system is easy and low-cost, but the flow rate can be unstable and slow for rapid solution exchange. Peristaltic pump perfusion offers stable and precise flow control, making it more appropriate for prolonged recordings and experiments requiring fast drug application.
  b. Adjust both inlet and outlet flow rates as needed.
    ***Note:*** Prevent liquid from leaking onto the microscope objective or stage to avoid damaging the equipment.
  c. Maintain an adequate liquid level to preserve brain slice viability and ensure proper immersion for the 60× objective and electrode tip.
  d. Prepare the pipette using for patch clamping.
    ***Note:*** Pull patch pipettes from borosilicate glass capillary tubing (BF150-110-10, 1.1-mm ID, 1.5-mm OD; Sutter instrument, Novato, CA) with a PC-10 puller (Narishige, Tokyo, Japan). Set pipette resistances to 2∼4 MΩ for recording pyramidal neurons and 4∼6 MΩ for astrocytes.
    **CRITICAL:** Adjust pipette resistance and tip geometry based on cell type. Use finer, higher-resistance pipettes for astrocytes to match their smaller size and minimize mechanical damage. Optimize pulling parameters to produce smooth, symmetrical taper and fine tips for high-resistance seals.
4. Turn on the equipment.
  a. Power on the Patch-Clamp 700B amplifier and 1550B digital-to-analog converter.
  b. Switch to the V-Clamp interface in 700B.
  c. Open the real-time imaging software.
  d. Transfer the brain slice to the recording chamber under the microscope (Figure 2A) and continuously perfuse it with oxygenated solution.

### Whole-cell patch clamping

**Timing: 10 min**

5. Whole-cell patch-clamp.
  a. Selecting Healthy Neurons.
    i. Acquire brain slice images using an upright infrared differential interference contrast (IR-DIC) microscope (Nikon, Japan) equipped with a 60× water-immersion objective.
    ii. Transmit the images to a monitor using a connected camera.
    iii. Identify healthy neurons by their transparent, light-colored appearance and clear cell edges.
      ***Note:*** Select only plump, well-conditioned neurons for patch clamping (Figure 2C). Avoid neurons that: 1) Appear darker in color, 2) Have visible cell edges or a clear nucleus, or 3) Show signs of shrinkage. In Figure 2C, the black arrows point healthy neurons, while the red arrow points to a damaged neuron.
  b. Electrode Preparation and Initial Setup.
    i. Open the Seal1 protocol in V-clamp mode (Figure 3A).

**Figure 3:**
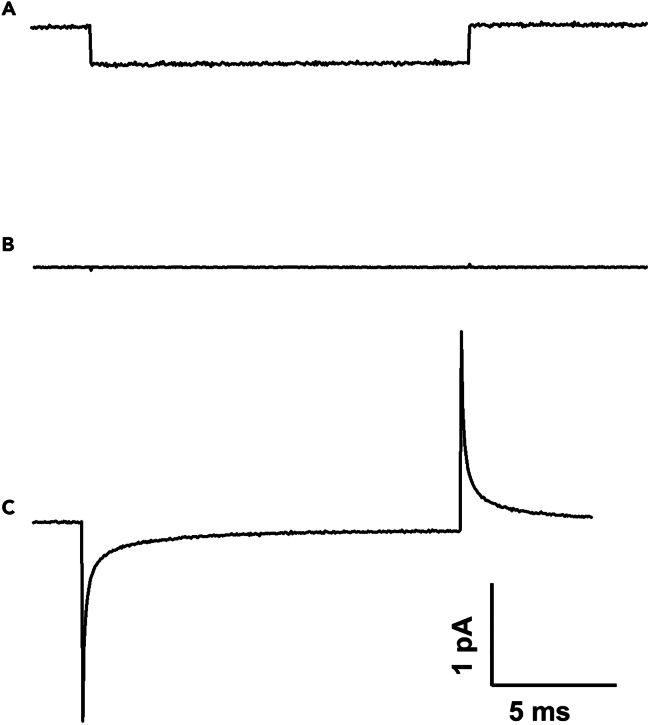
Electrode seal formation: From contact to GΩ-seal (A) Electrode in the aCSF solution with the pipette resistances around 4 MΩ. (B) Gigaseal Formation – Negative pressure applied; seal resistance rapidly increases, trace flattens. (C) Whole-Cell Access – Brief suction or zap breaks the membrane patch; whole-cell configuration established.
    ii. Fill the electrode halfway with intracellular solution.
    iii. Gently shake the electrode to remove air bubbles.
  c. Approaching the Neuron.
    i. Position the electrode tip in the solution.
    ii. Apply slight positive pressure to maintain airflow while lowering the electrode, preventing clogging.
      ***Note:*** Avoid excessive pressure to prevent disrupting seal formation or causing tissue damage.
    iii. Using 60× magnification to lower the electrode and objective lens toward the brain slice.
    iv. Reduce the descent speed as the electrode approaches the slice.
    v. Monitor the electrode movement on the screen.
  d. Forming High-Resistance Seal.
    i. Gently press the electrode tip against the neuron.
    ii. Observe a small dimple indicating contact with the cell membrane.
    iii. Apply brief negative pressure to create a high-resistance seal (GΩ), confirmed by a sharp increase in electrode resistance on the screen (Figure 3B).
  e. Gaining Whole-Cell Access.
    i. Switch to Seal 2 protocol.
    ii. Apply a rapid yet gentle negative pressure using either a syringe or mouth suction to rupture the membrane patch.
    iii. Break the cell membrane to achieve the whole-cell configuration for intracellular recordings (Figure 3C).
      ***Note:*** Avoid excessive force to prevent cell detachment or unstable break-in. Use mouth-controlled suction to allow gentle and precise rupture the membrane patch.
      **CRITICAL:** Use brain slices within 1–6 h after preparation. Perform recordings within the first 1–3 h after a 30-min incubation period, as neuronal viability and electrophysiological integrity gradually decline over time.
6. Seal protocol setting.
  a. Set up the Seal 1 protocol in voltage-clamp mode to form a high-resistance gigaseal (Table 2).
    i. 0 mV-step A stabilizes the electrode potential.
    ii. −1 mV-step B attracts the membrane to the electrode via electrostatic interaction.
    iii. Resistance increase indicates tight sealing around the electrode tip, preventing ion leakage.
      ***Note:*** During seal formation, the electrode resistance increases, and the trace transitions from a fluctuating or sloped line (Figure 3A) to an almost flat, horizontal line (Figure 3B), indicating minimal current flow and successful gigaseal formation (GΩ-seal). This high-resistance seal minimizes current leakage, ensuring stable recordings.

**Table 2:** Procedure for gigaseal formation

Procedure	Purpose	Voltage steps	Sample duration	Key function
Seal 1	Gigaseal formation	0 mV → −1 mV	50 samples (step A), 300 samples (step B)	Forms a high-resistance seal by ensuring tight membrane-electrode contact.
Seal 2	Membrane break-in	−70 mV → −80 mV	100 samples (step A), 600 samples (step B)	Induces controlled membrane rupture for whole-cell access.
  b. Once achieving a stable gigaseal, immediately switch to the Seal 2 protocol to gain whole-cell access (Table 2).
  c. Apply Seal 2 protocol to achieve membrane break-in and gain whole-cell recording.
    ***Note:*** This protocol applies a larger negative voltage to induce mechanical stress, causing the membrane to rupture, allowing whole-cell access. Rapid negative pressure helps to break the membrane (Figure 3C) while avoiding excessive force that could damage the cell or cause leakage.
7. Recording electrical signals.

Select the appropriate recording mode.

**Current-Clamp Mode:** Inject current to measure cell membrane potential changes, capturing action potentials and neuronal excitability.

**Voltage-Clamp Mode:** Hold the membrane voltage constant using the voltage-clamp technique to measure ionic currents across the cell membrane and analyze ion channel activity.

**I = 0Mode**: Use this model to test the resting membrane potential.

**CRITICAL:** Open the appropriate protocol file in Clampex according to the specific recording mode (e.g., current-clamp, voltage-clamp, or gap-free mode). These protocol files contain predefined settings including holding potentials, sampling rates, and stimulus waveforms optimized for each stage of the recording. For detailed step-by-step instructions on protocol setup and management, refer to *Setting Up Clampex Software for Data Acquisition by Rev. H, January 2021*. This document provides comprehensive guidance on configuring acquisition parameters, saving custom protocols, and organizing files for efficient recording setup.

### Electrophysiological signal recordings

**Timing: 4–5 h**

8. Whole-cell patch-clamp.
  a. Filter signals at 10 kHz (four-pole low-pass Bessel filter) and digitize at 20 kHz.
  b. Maintain the recording temperature at 30°C–32°C using an automatic temperature controller (TC-344C, Warner Instrument).
  c. For voltage-clamp recording, hole cells at −70 mV. After achieving whole-cell access, record the resting membrane potential (RMP) of cortical pyramidal neurons under I=0 mode.
  d. Examine basic electrophysiological properties (current-clamp mode)
    i. Inject stepwise current (starting at −240 pA, increasing by 30 pA or 20 pA).
    ii. Measure membrane voltage changes, input resistance (IR), and action potential firing patterns.
      ***Note:*** Calculate IR by dividing the voltage deflection in response to a −30 pA current injection by the amplitude of the injected current.
    iii. Analyze key electrophysiological parameters.
      ***Note:*** Determine the following metrics from the voltage responses:
      **Membrane time constant (τ)**—assess how quickly the membrane potential responds to a stimulus to reflect the membrane’s passive electrical properties.
      **Rheobase current—**identify the minimal current injection that elicits the first action potential to evaluate the excitability threshold.
      **Firing rate—**calculate the number of action potentials generated in response to a given current injection to assess the neuron’s firing behavior.
  e. Record synaptic activity (voltage-clamp mode, −70 mV).
    i. sEPSC: Record for 3–5 min.
    ii. mEPSC: Additional 1 μM TTX (Na^+^ channel blocker) ensures independence from action potentials.
      ***Note:*** Researchers commonly analyze the frequency of spontaneous EPSCs to indirectly assess presynaptic glutamate release activity, while also acknowledging that it also reflects the number of functional synapses. They interpret EPSC amplitude as an indicator of postsynaptic glutamate receptor responsiveness, although quantal size and synaptic location can also affect it. By evaluating both frequency and amplitude, researchers gain insights into both presynaptic and postsynaptic mechanisms underlying synaptic transmission.^27^
  f. Record evoked excitatory postsynaptic currents (eEPSCs).
    i. We use 400 μm-thick brain slices for eEPSC recordings.
    ii. Position the stimulation electrode within the targeted brain region in the slice.
    iii. Obtain a whole-cell recording using recording electrode in the target brain region.
    iv. Deliver stimulation pulses using a Master-8 stimulator connected to an A365 isolator.
    v. Adjust stimulation intensity to approximately 15 μA with a pulse duration of 0.2 s to evoke suitable evoked excitatory postsynaptic current (eEPSC) amplitudes.
    vi. Record eEPSCs at varying stimulation frequencies (e.g., 1 Hz, 5 Hz) to assess frequency response.
  g. Record inhibitory postsynaptic currents (IPSCs) (voltage-clamp mode, −70 mV).
    ***Note:*** Perform whole-cell patch-clamp recordings at 30°C–32°C to better reflect physiological conditions and more accurately capture intrinsic neuronal/cellular properties. Alternatively, use room temperature (23°C) recordings to minimize stimulation artifacts and prolong recording stability and brain slice viability.
9. Extracellular recording.
  a. Record compound action potentials (CAPs) in the corpus callosum (CC).
    ***Note:*** The CAP recordings in CC assess axonal conduction properties by stimulating nerve fibers and measuring population responses.^28^ These recordings provide insights into axonal excitability, conduction velocity, synaptic transmission, and drug effects. We record CAP of CC to evaluate axonal conduction in both myelinated and unmyelinated fibers by stimulating axonal tracts and recording population responses.
    i. Place the stimulating electrode on one side of CC to activate axonal fibers, while place recording electrode on the opposite side of CC to measure the resulting compound action potential.^28^
    ii. Record CAPs extracellularly using a glass electrode filled with extracellular solution in response to electrical stimulation.
      ***Note:*** Amplitude and latency of CAPs provide key insights into axonal conduction and synaptic efficiency.
  b. Record Long-term potentiation (LTP) in the hippocampus.
    Researchers commonly use long-term potentiation (LTP) to study synaptic plasticity. We use LTP to measure synaptic plasticity via field excitatory postsynaptic potential (fEPSP) recordings in the hippocampal CA1 region.
    i. Position the recording electrode in the CA1 region of the hippocampus, where Schaffer collateral inputs terminate.
    ii. Place the stimulating electrode in the Schaffer collateral pathway (CA3-CA1 synapse) (Figure 6A).
    iii. Deliver test stimuli (0.033 Hz) and record baseline fEPSP responses.
    iv. Adjust the stimulation intensity to evoke 40–50% of the maximum fEPSP response.
    v. Record a stable baseline for 15–30 min.
      ***Note:*** We typically increase the stimulus intensity gradually to observe the corresponding change in the evoked response.
    vi. Induce LTP using a high-frequency stimulation (HFS) protocol: apply 100 Hz stimulation for 2 s (repeated 2–3 times with a 10-s interval).^29^^,^^30^
    vii. Continue recording fEPSP responses for minimum 60 min after induction.
    viii. Measure and analyze fEPSP slope and peak amplitude as percentage of baseline values.
      **CRITICAL:** Identify successful LTP by a sustained increase in fEPSP amplitude (∼120% or higher of baseline) maintained for at least 60 min after HFS.
      ***Note:*** Long-term potentiation (LTP) is a key indicator of synaptic plasticity and involves both AMPA and NMDA receptors. Record AMPA receptor-mediated LTP at a holding potential of −70 mV. In contrast, NMDA receptor-dependent LTP requires depolarization (e.g., +40 mV) and often removal of Mg^2+^ from the extracellular solution to relieve the voltage-dependent block of NMDA receptors.^5^^,^^31^ Use field recordings (fEPSP) to capture overall synaptic responses primarily driven by AMPARs, while whole-cell patch-clamp allows precise control of membrane potential to isolate AMPAR or NMDAR currents.

## Expected outcomes

Astrocytes, a major type of glial cell in the CNS, play essential roles in maintaining brain homeostasis, supporting neurons, and regulating synaptic function, actively influencing neuronal activity from individual synapses to large-scale networks.^32^ In our previous research, we indicated that the astroglia Sox2 regulate cortical-striatal mEPSCs in the mouse.^1^ Characterizing the electrophysiological properties of astrocytes, in parallel with pyramidal neurons, can provide complementary information for understanding the cellular environment and potential mechanisms of neuron-glia interactions. In this protocol, we perform electrophysiological recordings of pyramidal neurons and astrocytes to characterize their intrinsic membrane properties which help clarify the physiological basis for understanding how astrocytic networks modulate neuronal activity and circuit function.

### Whole-cell patch clamp of pyramidal neurons and astrocytes

**Figure 4:**
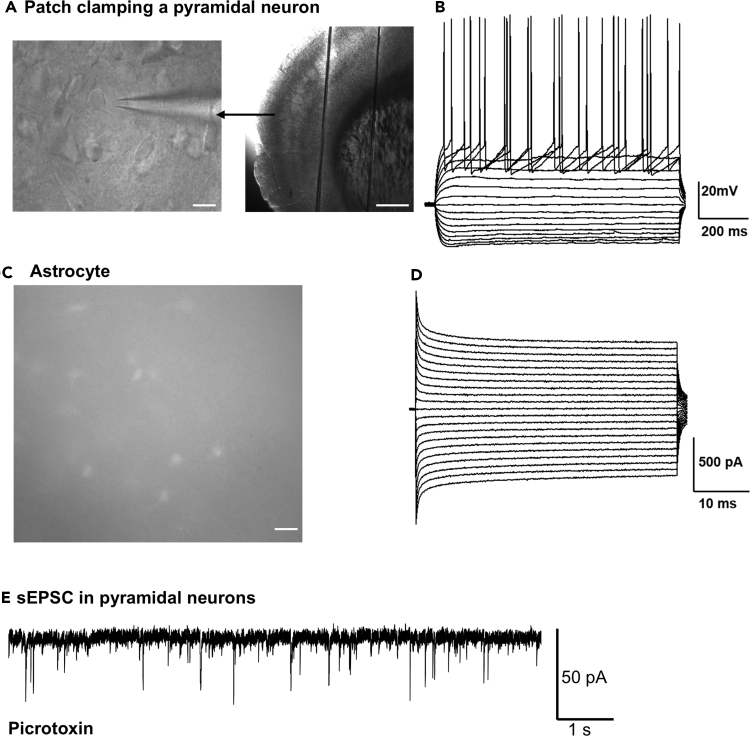
Patch-clamp recording of pyramidal neurons and astrocytes (A) Cortical pyramidal neuron under a 60× objective during patch-clamp recording. Scale bar represents 15 μm and 0.5 mm respectively. (B) Depolarizing current injection in a pyramidal neuron. (C) Cortical astrocyte labeled with SR101. Scale bar represents 10 μm. (D) Voltage-step response (10 mV) in a cortical astrocyte. (E) Raw trace of sEPSCs in a cortical pyramidal neuron.

We perform whole-cell patch-clamp recordings in acute brain slices (PN25, C57BL/6 mice) to characterize the intrinsic membrane properties of cortical pyramidal neurons and astrocytes. We inject current pulses into pyramidal neurons to induce membrane potential changes, measure input resistance, and evoke action potentials (Figure 4B). The amplitude of the current pulses starts at −240 pA and increase in 30 pA increments. We use voltage-clamp recordings with 10 mV step increments to assess astrocyte membrane currents (Figure 4D). Calculate input resistance (IR) using Ohm’s law. Determine input resistance (IR) in pyramidal neurons by measuring the voltage change induced by a −30 pA current injection. Measure IR in astrocytes by applying 10 mV step changes and analyzing the resulting steady-state current.

Astrocytes exhibit distinct membrane properties, such as resting membrane potential (RMP) and input resistance, compared with neurons. We list the averages of membrane properties of astrocyte and pyramidal neuron (PN) in Table 3. Astrocytes exhibit more hyperpolarized RMP compared with pyramidal neurons (astrocytes: −79.09 ± 0.6 mV, n = 16 cells, PN: −75.72 ± 0.2 mV, n =18 cells). The highly negative RMP of astrocytes helps them buffer extracellular K^+^ levels, preventing excessive neuronal excitability. Astrocytes also have lower input resistance (19.52 ± 0.34 MΩ) compared with pyramidal neurons (120.3 ± 1.3 MΩ), primarily due to the high expression of potassium leak channels, including two-pore domain K^+^ channels.^33^^,^^34^ We also measure changes in membrane potential during depolarizing current injections in both astrocytes and pyramidal neurons in current clamp mode. Unlike pyramidal neurons, which generate action potentials during depolarizing current injections (5.1 ± 0.5 spikes at 180 pA), astrocytes exhibit passive membrane properties without spiking.

**Table 3:** Electrophysiological properties of motor cortical layer II/III pyramidal neurons and astrocytes

	Cortical pyramidal neuron	Cortical astrocyte
RMP (mV)	−75.72 ± 0.2	−79.09 ± 0.6
Input resistance (MΩ)	120.3 ± 1.3	19.52 ± 0.34
Membrane t (ms)	17.4 ± 0.4	3.67 ± 0.25
Spike no. evoked by 180 pA	5.1 ± 0.5	No

Values are mean ± SEM. Spike no.: Number of spikes induced by 180 pA current injection (PN: n = 18 cells, Astrocytes: n = 16 cells).

### Synaptic transmission in cortical pyramidal neurons

In addition to measure basic electrophysiological properties of neuronal/astroglial membrane, we use whole-cell patch-clamp to investigate synaptic transmission by recording various excitatory and inhibitory postsynaptic currents (EPSCs and IPSCs, eEPSC). Researchers use EPSCs and IPSCs to assess the excitation and inhibition balance in neural circuits, providing a more comprehensive understanding of synaptic transmission and its role in neuronal function and network activity. Evoked EPSCs provides insights into synaptic strength, presynaptic release probability, short-term plasticity, and the impact of GABAergic inhibition on excitatory transmission.

We use Mini analysis software to analyze the amplitude and frequency of sEPSC in cortical pyramidal neurons (Figure 4E). Pyramidal neuronal sEPSCs frequency or amplitude may be different in different regions in the brain,^35^ also the ages of the mice matter.^36^^,^^37^ In our recorded neurons of PN 25 days mice, the amplitude of sEPSC is 20.79 ± 0.2 pA, and the frequency of sEPSC is 4.40 ± 0.09 Hz (n =18 cells).

### Extracellular recording

Extracellular recordings are essential for studying neural circuit functions and plasticity under normal and pathological conditions. We use CAPs and LTP as examples to delineate extracellular recording techniques and data analyses.

### CAP recordings

**Figure 5:**
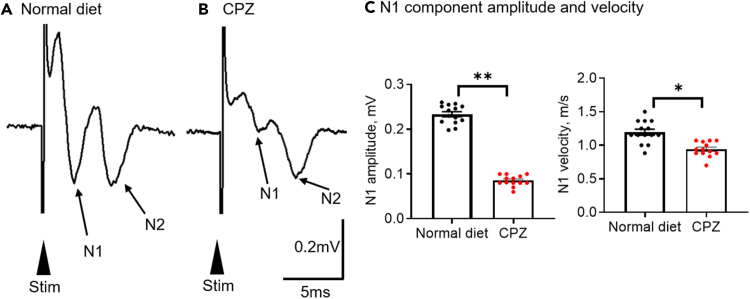
The impaired nerve transmission in CC during CPZ-induced demyelination (A and B) Examples of CAPs recorded in CC in normal diet and CPZ feeding for 4 weeks. (C) Summary of the amplitudes and velocity of the N1 component, Student’s t-test, ∗∗p < 0.01, ∗p < 0.05.

CAP recordings provide a reliable tool for evaluating the functional integrity of myelin. Unlike whole-cell patch-clamp, extracellular recordings of CAPs capture large-scale network activity. Myelination plays a crucial role in enhancing nerve signal transmission efficiency. To assess axonal conduction changes following demyelination, we record CAPs in corpus callosum (CC) of 2 month-old C57BL/6 mice treated with 1 month of normal and cuprizone (CPZ) diet which elicits demyelination in the CC.^38^ As shown in Figure 5, four weeks of CPZ treatment significantly reduce the amplitude and conduction velocity of the fast N1 component of CAPs, which myelinated axons primarily mediate, compared with control mice fed a normal diet. These results indicate that CPZ-induced demyelination impairs nerve conduction in the CC.

### LTP induction

LTP is a key model for studying synaptic plasticity, crucial for learning and memory. Induce LTP reliably using high-frequency stimulation (HFS) or theta burst stimulation (TBS). Researchers typically apply high-frequency stimulation (HFS) using brief trains of stimuli at gamma frequency (100 Hz for 1 s), a method extensively validated in both in vivo and in vitro studies.^39^^,^^40^ TBS mimics endogenous theta rhythms by delivering bursts of high-frequency stimuli at theta intervals (5–7 Hz), also produces robust LTP.^41^

**Figure 6:**
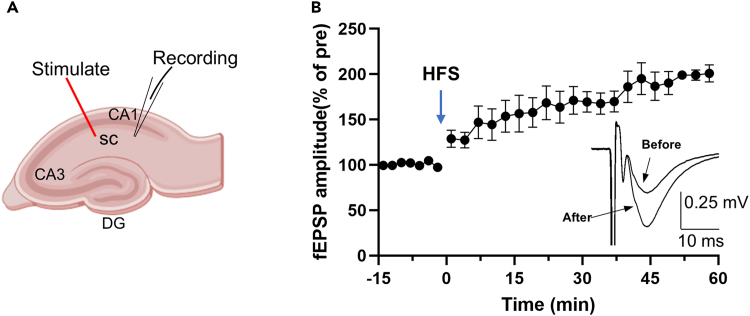
LTP recording in hippocampus (A) Schematic of a hippocampal slice showing stimulating (stim) and recording (record) sites. SC, Schaffer collaterals; DG, Dentate gyrus. (B) Induce LTP by high-frequency stimulation (HFS) in hippocampus.

To evaluate LTP in the hippocampus, we perform extracellular field recordings in acute slices, measuring field excitatory postsynaptic potentials (fEPSPs) before and after high-frequency stimulation (HFS). Baseline recordings ensure stable synaptic responses, while post-HFS recordings assess LTP induction and maintenance over 45 min. Elicit test responses at 0.033 Hz. Induce LTP using HFS protocol (100 Hz for 2 s, three trains, 10 s inter-train interval),^42^ leading to a sustained increase (60 min) in synaptic strength. As shown in Figure 6B, representative fEPSP traces and statistical analyses confirm successful LTP induction in hippocampus, illustrating synaptic strength changes and plasticity mechanisms.

## Quantification and statistical analysis

We use MultiClamp 700B amplifier, pClamp 9.2 software, and Digidata 1550B interface (Molecular Devices) to acquire data. Exclude cells in which access resistance increases by more than 15% from data collection. Analyze electrophysiological data using Clampfit 9 (Molecular Devices, CA). We present all data in mean ± SEM. Analyze spontaneous synaptic current data using Mini Analysis software (Synaptosoft, Fort Lee, NJ, USA). Use Axon Clampfit to analyze synaptic events with both threshold and template-based searches as an alternative to the Mini Analysis program. Perform comparisons between two groups using Student’s t-test. Use two-way ANOVA or one-way ANOVA to compare changes among different groups, followed by Tukey post hoc tests. Use the Kolmogorov-Smirnov (K-S) test to compare cumulative distributions of sEPSCs.

## Limitations

This protocol offers a detailed workflow for whole-cell and extracellular electrophysiology recordings; however, we acknowledge several limitations. First, while acute brain slices preserve local neural circuits, they lack long-range connectivity and do not fully replicate in vivo physiological conditions. Second, slice quality, equipment stability, and, most critically, the operator’s technical skill greatly influence the reliability and reproducibility of the data. A major limitation of this protocol is that acquiring high-quality electrophysiological recordings requires substantial hands-on experience for new users. Furthermore, accurate data interpretation demands a solid understanding of the underlying molecular and cellular mechanisms, which may pose a challenge for less-experienced users.

In the troubleshooting section, we have highlighted only the most common and critical issues. However, a wide range of case-by case problems may arise throughout the experimental process, including signal acquisition failure, undetectable responses, and instrument-related malfunctions, which we do not discuss in detail here. We encourage users to consult the relevant equipment manuals for further troubleshooting guidance. Additional unforeseen issues may also occur and should be addressed based on the specific experimental context.

## Troubleshooting

### Problem 1

Poor GΩ seal formation.

High-resistance (GΩ) seal formation is a critical step in successful whole-cell patch-clamp recordings and one of the most common technical challenges (Step 5d).

### Potential solution

Several factors can contribute to seal failure.

• Pipette tip condition: Ensure that the pipette tip is clean and smooth, with an appropriate resistance (typically 2∼4 MΩ for neurons) (Step 3d).
• Pressure control: Maintain positive pressure while approaching the cell with the pipette to prevent clogging, but avoid applying excessive pressure that could damage the cell (Step 5b). As the pipette nears the membrane, apply a brief, controlled negative pressure to promote seal formation (Step 5d). Excessive suction may rupture the membrane, while insufficient suction may fail to form a stable seal (Step 5e).
• Slice and cell quality: The most important part of successful seal formation is the health of the brain slice. Ensure rapid brain extraction and slicing under low temperature, oxygenated cutting solution to preserve tissue viability (Step 2). Under the microscope, select only plump, well-conditioned neurons for whole-cell patch-clamping (Figure 2C) (Step 5a).

### Problem 2

Difficulty in achieving whole-cell access (Step 5e).

### Potential solution

• Electrode positioning: When approaching the target cell, position the pipette tip near the center of the soma rather than at the periphery to increase the likelihood of forming a high-resistance seal (Step 5c).
• Avoid excessive positive pressure during approach, as this can rupture the membrane prematurely, resulting in unstable sealing or complete cell detachment (Step 5b).
• Visual feedback and suction application: As the pipette slowly approaches the cell membrane, monitor under DIC or IR-DIC microscopy for visible membrane indentation and a small meniscus or dimple at the pipette tip (Step 5c). At this moment, rapidly apply a brief negative pressure via the syringe to initiate seal formation.
• Check for air leaks: Ensure that all connections in the pressure line—especially the tubing junctions and the pipette holder assembly—are airtight. Any leakage will compromise the ability to apply precise positive or negative pressure, thereby reducing the success rate of seal formation.

### Problem 3

Difficulty in LTP induction (Step 9b).

### Potential solution

During LTP recordings, precise positioning of both the stimulating and recording electrodes is critical for obtaining reliable synaptic responses (Step 9b). If the evoked field excitatory postsynaptic potential (fEPSP) is absent or subthreshold, adjust the location of either the stimulating or the recording electrode, but not both simultaneously, to optimize signal detection and maintain spatial resolution. Inspect the stimulating electrode tip for structural integrity and avoid excessive tissue adhesion, as this can attenuate or disrupt current delivery. Additionally, verify the operational status of the stimulus isolator; a depleted battery can result in a failure to deliver current. The presence or absence of a stimulus artifact in the recording trace can serve as a diagnostic indicator of whether stimulation is occurring.

## Resource availability

### Lead contact

Further information and requests for resources and reagents should be directed to and will be fulfilled by the lead contact, Dr. Fuzheng Guo (fzguo@health.ucdavis.edu).

### Technical contact

Huimin Chen (hmichen@health.ucdavis.edu).

### Materials availability

This study did not generate new unique reagents.

### Data and code availability

This study did not generate or analyze datasets or code.

## Acknowledgments

This work was funded by 10.13039/100000002NIH/10.13039/100000065NINDS (R21NS125464, R01NS123080, R01NS123165, and R01NS134887) and 10.13039/100011781Shriners Hospitals for Children (85113-NCA-23).

## Author contributions

Conceptualization, H.C. and F.G.; investigation, H.C. and X.S.; writing, H.C.; review and editing, F.G.; funding acquisition, F.G.

## Declaration of interests

The authors declare no competing interests.
